# Use of simple scoring systems for a public health approach in the management of non‐alcoholic fatty liver disease patients

**DOI:** 10.1002/jgh3.12414

**Published:** 2020-09-12

**Authors:** Shamsul Mohd Zain, Hwa‐Li Tan, Zahurin Mohamed, Wah‐Kheong Chan, Sanjiv Mahadeva, Roma Choudhury Basu, Rosmawati Mohamed

**Affiliations:** ^1^ Department of Pharmacology, Faculty of Medicine University of Malaya Kuala Lumpur Malaysia; ^2^ Department of Medicine, Faculty of Medicine University of Malaya Kuala Lumpur Malaysia; ^3^ Clinical Investigation Centre University Malaya Medical Centre Kuala Lumpur Malaysia

**Keywords:** advanced fibrosis, fatty liver, FIB‐4, non‐alcoholic fatty liver disease, scoring system

## Abstract

**Background and Aim:**

Advanced fibrosis is the most important predictor of liver‐related mortality in non‐alcoholic fatty liver disease (NAFLD). The aim of this study was to compare the diagnostic performance of noninvasive scoring systems in identifying advanced fibrosis in a Malaysian NAFLD cohort and propose a simplified strategy for the management of NAFLD in a primary care setting.

**Methods:**

We enrolled and reviewed 122 biopsy‐proven NAFLD patients. Advanced fibrosis was defined as fibrosis stages 3–4. Noninvasive assessments included aspartate aminotransferase/alanine aminotransferase (AST/ALT) ratio, AST‐to‐platelet ratio index (APRI), AST/ALT ratio, diabetes (BARD) score, fibrosis‐4 (FIB‐4) score, and NAFLD fibrosis score.

**Results:**

FIB‐4 score had the highest area under the receiver operating characteristic curve (AUROC) and negative predictive value (NPV) of 0.86 and 94.3%, respectively, for the diagnosis of advanced fibrosis. FIB‐4 score < 1.3 ruled out advanced fibrosis in 72% of the patients, with 6% being understaged. Further stratification of the indeterminate group patients by other non‐alcoholic steatohepatitis (NASH) clinical predictors, such as abnormal gamma‐glutamyl transpeptidase (GGT) level and presence diabetes mellitus (DM), could further reduce the number of patients who are unlikely to have advanced fibrosis by 52% and 35%, respectively.

**Conclusion:**

We found that FIB‐4 score outperforms other scoring systems based on AUROC and NPV. The use of a simple scoring system such as FIB‐4 as first‐line triage to risk‐stratify NAFLD patients in the primary care setting, with further stratification of those in the indeterminate group using clinical predictors of NASH, can help in the development of a simplified strategy for a public health approach in the management of NAFLD.

## Graphical abstract

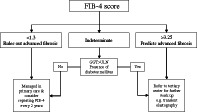

The aim of this study was to compare the diagnostic performance of non‐invasive scoring systems in identifying advanced fibrosis in a Malaysian NAFLD cohort and propose a simplified strategy in the management of NAFLD in a primary care setting. FIB‐4 score outperforms other scoring systems based on AUROC and NPV. Further stratification of the patients within the indeterminate group by other clinical predictors of NASH such as abnormal GGT level and presence diabetes mellitus (DM) could further reduce the number of patients who are unlikely to have advanced fibrosis by 52% and 35% for DM, respectively.

## Introduction

Non‐alcoholic fatty liver disease (NAFLD) is the most common liver disorder in Western countries, affecting 17–46% of adults, and accounts for 26.4% of the patients with abnormal liver test in a United Kingdom‐based community study.^1^ NAFLD is the hepatic manifestation of the metabolic syndrome with increasing prevalence worldwide, paralleling the epidemic of obesity and diabetes, with the global estimated prevalence at 24% and 27% in Asia, respectively.^2^ Patients with NAFLD have a higher mortality rate compared with the general population, mainly attributed to cardiovascular disease, malignancy, or liver‐related mortality.^3^ NAFLD has emerged as one of the century's imminent public health problems. The apparent challenge, however, is that the disease is often asymptomatic until the late stage of NAFLD.^4^ Among NAFLD patients, those with advanced fibrosis (stages 3 and 4) exhibit the worst prognosis independent of their NAFLD Activity Score (NAS), with hazard ratio ranging from 3.3 to 5.7.^5^ It is therefore imperative to identify subgroups of patients with NAFLD that are at high risk of adverse outcomes and will require additional workup and surveillance so that interventions can be targeted to patients at greatest need.

As NAFLD is often discovered incidentally based on elevated liver enzymes in the primary care setting, the next step would be to risk‐stratify the patients prior to referral to secondary or tertiary care, with a focus on the presence of advanced fibrosis. The current gold standard of liver biopsy offers a vast array of information, including the degree of steatosis, severity of necroinflammation, hepatocellular ballooning, and fibrosis stage.^6^, ^7^ Notwithstanding that, liver biopsy, being an invasive procedure, has other limitations, such as sampling error, that are not acceptable or practical for longitudinal monitoring of fibrosis progression, and it is not feasible to be performed on all NAFLD patients.^8^

This has led to the emergence of several noninvasive scoring systems utilizing anthropometric data and easily available clinical parameters such as aspartate aminotransferase/alanine aminotransferase (AST/ALT) ratio, AST‐to‐platelet ratio index (APRI), body mass index (BMI), AST/ALT ratio, diabetes (BARD) score, fibrosis‐4 (FIB‐4) score, and NAFLD fibrosis score (NFS). They have been validated against liver biopsy with variable accuracy in different populations with the capability to identify or rule out advanced fibrosis in NAFLD patients.^9^ As these scoring methods are calculated based on simple clinical parameters, they are easy to be carried out on a routine basis without incurring a huge cost.

The aim of this study was to compare and validate the diagnostic accuracy and clinical utility of noninvasive tests, including AST/ALT ratio, APRI, BARD score, FIB‐4 score, and NFS, in a cohort of biopsy‐proven NAFLD patients. As Malaysia is one of the most obese countries in Asia,^10^ this study can help to inform policymakers of a simplified strategy for a public health approach, particularly in the community setting, to identify those at risk of liver‐related mortality. Furthermore, there is a limited number of studies in Asia, and to the best of our knowledge, this is the first study that further suggests the need to stratify the indeterminate group of patients by assessing their clinical parameters, such as serum gamma‐glutamyl transpeptidase (GGT) level and presence of diabetes mellitus. This approach helps to avoid a substantial number of indeterminate patients from being referred to secondary/tertiary care settings.

## Methods

### *Patients*

Consecutive recruitment of 122 adult NAFLD patients from the University of Malaya Medical Centre (UMMC) from 2009 until 2014 was based on initial increase in liver echogenicity compared to renal cortex on ultrasound examination. Liver biopsy confirmation was performed, and histological grading was as recommended by the NASH Clinical Research Network.^7^ Advanced fibrosis was defined as samples with a fibrosis score of 3 or 4. We excluded patients with alcohol intake >21 units per week for men and >14 units per week for women^11^ and those with coexisting liver disease such as autoimmune hepatitis, chronic viral hepatitis, Wilson's disease, primary biliary cirrhosis, hemochromatosis, α1‐antitrypsin deficiency, biliary obstruction, and drug‐induced liver steatosis. Written informed consent was obtained, and the study protocol was approved by the Medical Ethics Committee of UMMC. The sample size was calculated using the formula for a cross‐sectional study, *n* = [(z^2^ * p * q)]/d^2^.^12^ Assumptions were made based on the Gut and Obesity in Asia Workgroup: p = prevalence of advanced fibrosis 24%, z for 95% confidence interval = 1.96, and d = error ≤ 10%. A sample size of 70 participants was estimated.

### *Clinical evaluation and biochemistry profiling*

Anthropometric measurements were taken, including body weight (kg), body height (m), and waist circumference (cm). Body mass index (BMI) was calculated as weight divided by height squared (kg/m^2^). Fasting blood samples were taken, and standard measurement of serum ALT, AST, GGT, low‐density lipoprotein (LDL)‐cholesterol, high‐density lipoprotein (HDL)‐cholesterol, total cholesterol, triglycerides, and albumin was carried out using Advia 2400 (Siemens Healthcare Diagnostics Inc., Deerfield, IL, USA) at the designated laboratory in UMMC. Whole‐blood measurement of the presence of hemoglobin A1c (HbA1c) (%) and platelet count (x10^9^/L) was also carried out. The AST/ALT ratio, APRI, BARD score, FIB‐4 score, and NFS were calculated accordingly based on the anthropometric measurements and biochemistry profiles. The calculation for AST/ALT ratio follows AST (IU/L)/ALT (IU/L),^13^ APRI follows AST (IU/L)/(upper limit of normal)/platelet count (10^9^/L) × 100,^14^ BARD score follows weighted sum of three variables (BMI ≥28 = 1 point, AST/ALT ratio ≥ 0.8 = 2 points, diabetes =1 point),^15^ FIB‐4 score follows age (year) × AST (IU/L)/platelet count (x10^9^/L) × √ALT (IU/L),^16^ and NFS follows −1.675 + 0.037 × age (year) + 0.094 × BMI (kg/m^2^) + 1.13 × impaired fasting glucose/diabetes (yes = 1, no = 0) + 0.99 × AST/ALT ratio‐0.013 × platelet (x10^9^/L)‐0.66 × albumin (g/dL).^17^

### *Statistical analysis*

All statistical analyses were performed using SPSS version 16.0 (IBM Corp, Chicago, IL, USA). Data were expressed in mean ± standard deviation (SD) for normally distributed continuous data, median (interquartile range) for nonnormally distributed continuous data, and percentage for categorical data. The comparison of two groups used independent *t*‐test and Mann–Whitney U for normally distributed and skewed variables, respectively. Categorical data were compared using the Chi square (χ2) test. The diagnostic accuracy of the noninvasive scoring systems was calculated using the area under receiver operating characteristics (AUROC) curve, and the 95% confidence interval (CI) was determined. The sensitivity, specificity, positive predictive value (PPV), and negative predictive value (NPV) were calculated based on the cut‐off values in the previously published reports.

## Results

We reviewed a total of 122 NAFLD patients. The mean age of the patients at time of liver biopsy was 50.0 (±11.4 SD) years. BMI ≥25 kg/m^2^ ^18^ was observed in 85% of the patients, and 48% of the patients were diabetic. All patients were included in the clinical scoring tests. NAFL was found in 25 patients (20%), while 97 patients (80%) had non‐alcoholic steatohepatitis (NASH). Thirty‐nine patients (32%) had fibrosis score = 0, 53 patients (43%) had fibrosis score = 1, 6 patients (6%) had fibrosis score = 2, and 24 patients (20%) had advanced fibrosis or cirrhosis (Kleiner fibrosis score = 3–4). As the scoring systems aim to identify patients with advanced fibrosis, the clinical and biochemistry parameters of patients with no/mild fibrosis (fibrosis = 0–2) were compared against patients with advanced fibrosis (stage 3–4), as shown in Table 1. Patients with advanced fibrosis were significantly much older (*P =* 0.001) with greater levels of HbA1c (*P =* 0.003), AST (*P =* 0.001), and GGT (*P* < 0.0001) but lower levels of LDL‐cholesterol (*P =* 0.012), total cholesterol (*P =* 0.042), and platelet count (*P =* 0.002).

**Table 1:** Demographic and clinical data of the NAFLD patients

Characteristics	*n* (%) or Mean ± SD or Median (Interquartile range)		*P‐*value
	Fibrosis 0–2 (*n* = 98)	Fibrosis 3–4 (*n* = 24)	
Gender			
Males	51 (52)	10 (42)	0.362
Females	47 (48)	14 (58)	
Age (years)	51.0 (17.0)	59.0 (10.0)	0.001
BMI (kg/m^2^)	28.6 (4.7)	29.5 (6.0)	0.475
HbA1c (%)	5.9 (1.0)	6.9 (1.8)	0.003
Diabetes	41 (42)	17 (71)	0.011
Waist circumference (cm)	95.0 (12.4)	98.9 (16.5)	0.062
HDL‐cholesterol (mg/dL)	46.0 (12.9)	46.4 (14.7)	0.629
LDL‐cholesterol (mg/dL)	122.8 ± 3.8	101.3 ± 34.7	0.012
Total cholesterol (mg/dL)	196.4 ± 43.7	176.3 ± 38.9	0.042
Triglycerides (mg/dL)	141.7 (70.9)	128.4 (50.9)	0.230
Albumin (d/dL)	4.3 ± 0.3	4.1 ± 0.4	0.054
Platelet (×10^9^/L)	278.6 ± 64.4	234.7 ± 48.8	0.002
AST (IU/L)	38.0 (31.0)	57.5 (39.0)	0.001
ALT (IU/L)	66.5 (63.0)	77.0 (74.0)	0.228
GGT (IU/L)	71.0 (74.0)	116.0 (129.0)	<0.0001
AST/ALT ratio	0.6 (0.2)	0.7 (0.2)	0.005
APRI	0.4 (0.4)	0.7 (0.5)	<0.0001
BARD score	1.0 (1.0)	2.0 (2.0)	0.001
FIB‐4 score	0.8 (0.6)	1.7 (0.7)	<0.0001
NFS	−2.5 ± 1.2	−1.0 ± 1.1	<0.0001

Data are expressed in mean ± SD for normally distributed continuous data, median (interquartile range) for nonnormally distributed continuous data, and percentage for categorical data.

ALT, alanine transferase; APRI, aspartate aminotransferase‐to‐platelet ratio index; AST, aspartate aminotransferase; BARD, BMI, AST/ALT ratio, diabetes; BMI, body mass index; FIB‐4, Fibrosis‐4; GGT, gamma‐glutamyl transpeptidase; HbA1c, hemoglobin A1c; HDL, high‐density lipoprotein; LDL, low‐density lipoprotein; NFS, NAFLD fibrosis score.

**Figure 1:**
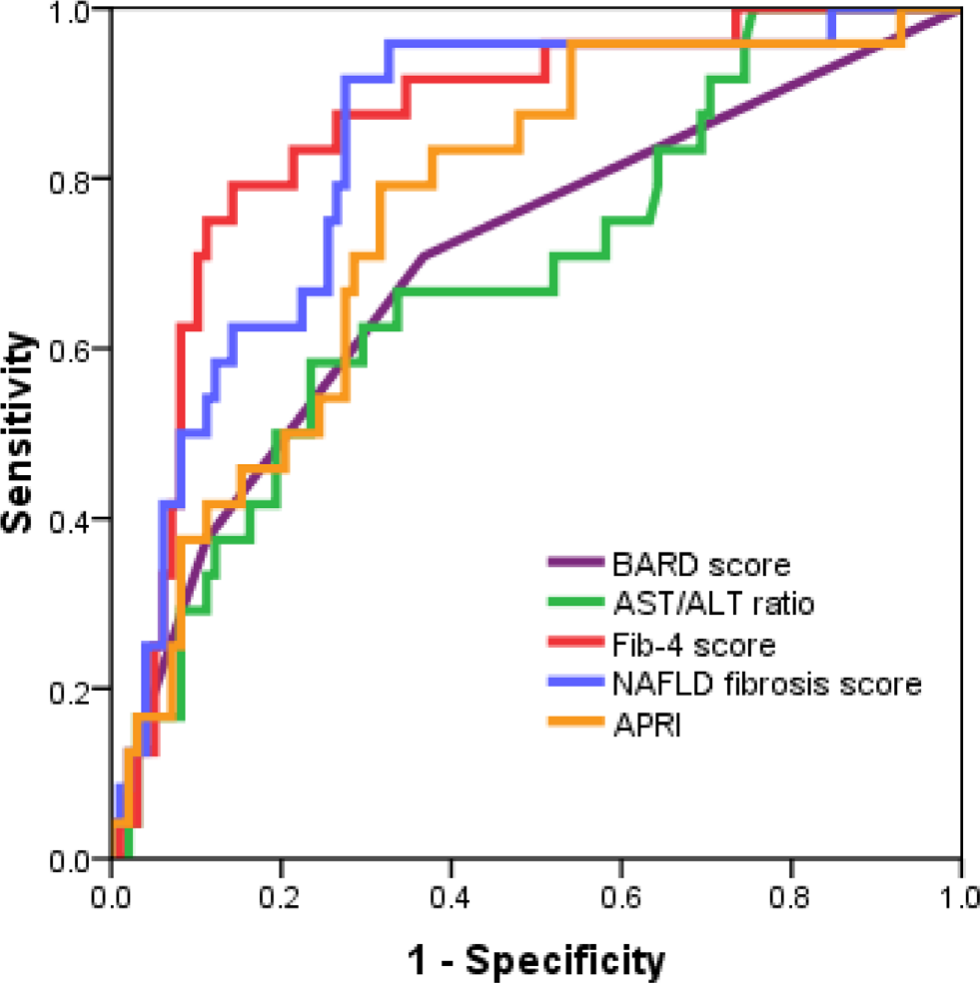
Receiver operating characteristic (ROC) curves for the noninvasive scores for the diagnosis of advanced fibrosis (stages 3–4).

The AUROC, sensitivity, specificity, NPV, and PPV were calculated to compare the diagnostic performance of the scoring systems (Table 2, Fig. 1). The AUROC ranged from 0.69 to 0.86. FIB‐4 score had the best AUROC (0.86) followed by NFS (0.84), APRI (0.76), BARD score (0.70), and AST/ALT ratio (0.69). All tests recorded high NPV > 80% using the lower cut‐off values, with the highest seen in FIB‐4 score and APRI (94%). The BARD score and NFS also performed well with an NPV of 90% and 89%, respectively. Nonetheless, the PPVs were modest, ranging from 32% to 56%. Table 3 demonstrates that a significant proportion of patients could avoid liver biopsy. FIB‐4 score outperformed others, with 72% of the patients who could avoid liver biopsy and only 6% of the patients misclassified. We also observed that a relatively high number of patients could avoid biopsy for AST/ALT ratio (81%) and NFS (70%), but the false negative results (15% and 11%, respectively) need to be considered.

**Table 2:** Comparison of the performance of noninvasive scores for the diagnosis of advanced fibrosis in NAFLD patients

Advanced fibrosis						
Test	AUROC (95% CI)	Cut‐off	Sensitivity (%)	Specificity (%)	PPV (%)	NPV (%)
AST/ALT ratio	0.687 (0.57–0.80)	0.8	37.5	83.7	36.0	84.5
		1	16.7	91.8	33.3	81.8
APRI	0.759 (0.66–0.86)	0.5	83.3	59.2	33.3	93.5
		1	37.5	91.8	52.9	85.7
BARD score	0.702 (0.58–0.82)	2	70.8	63.3	32.1	89.9
FIB‐4 score	0.857 (0.78–0.94)	1.3	79.2	84.7	55.9	94.3
		3.25	4.2	98.0	33.3	80.7
NFS	0.836 (0.75–0.92)	−1.455	62.5	77.6	40.5	89.4
		0.676	4.2	99.0	50.0	80.8

ALT, alanine transferase; APRI, aspartate aminotransferase‐to‐platelet ratio index; AST, aspartate aminotransferase; AUROC, area under receiver operating characteristics; BARD, BMI, AST/ALT ratio, Diabetes; CI, confident interval; FIB‐4, Fibrosis‐4; NFS, NAFLD fibrosis score; NPV, negative predictive value; PPV, positive predictive value.

**Table 3:** Number of patients avoiding liver biopsy

Test	Cut‐off	Patients avoiding referral	False negative result
AST/ALT ratio	0.8	99/122 (81%)	15 (15%)
APRI	0.5	62/122 (51%)	4 (6%)
BARD score	2	69/122 (57%)	7 (10%)
FIB‐4 score	1.3	88/122 (72%)	5 (6%)
NFS	−1.455	85/122 (70%)	9 (11%)

*ALT*, alanine transferase; *APRI*, aspartate aminotransferase‐to‐platelet ratio index; *AST*, aspartate aminotransferase; *BARD*, BMI, AST/ALT ratio, Diabetes; *FIB‐4*, Fibrosis‐4; *NFS*, NAFLD fibrosis score.

**Figure 2:**
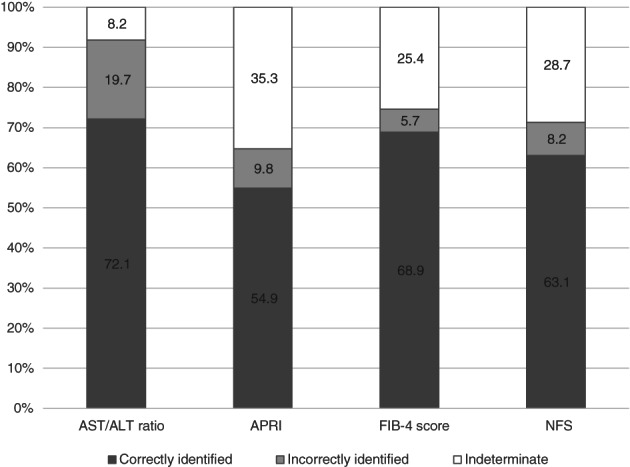
Utility of noninvasive panels with dual cut‐off values.

Utility of dual cut‐off values also suggests the FIB‐4 score to be superior compared to other tests (Fig. 2). FIB‐4 score offers dual cut‐off values whereby the low published cut‐off of less than 1.3 ruled out advanced fibrosis, whereas a score of greater than 3.25 predicts advanced fibrosis. In this study, using a low published cut‐off of 1.3 for FIB‐4 score, we not only recorded a high number of patients correctly identified (68.9%) but also present the lowest number of misclassified patients (5.7%). About 25% (*n* = 31) of the patients were in the indeterminate range, whereby 18 patients had advanced fibrosis, and 13 patients did not. We further stratified the patients within the indeterminate group by other clinical predictors of NASH, such as serum GGT level above upper limit normal (ULN) and presence of diabetes mellitus.^19^ We then evaluated the magnitude of effects of these clinical predictors on advanced fibrosis risk. We found that patients with serum GGT level above ULN are associated with 13‐fold increased risk (OR 12.80, 95% CI 2.02–81.12, *P* = 0.007), while the presence of diabetes mellitus was associated with a 5‐fold (OR 5.24, 95% CI 1.06–25.97, *P* = 0.043) increased risk of advanced fibrosis. Further stratification of the patients within the indeterminate group by serum GGT level above ULN and presence of diabetes mellitus could further reduce the number of patients who are unlikely to have advanced fibrosis by 52% (*n* = 16/31) and 35% (*n* = 11/31), respectively. Copresence of these clinical predictors increases the risk of advanced fibrosis by 19‐fold (OR 18.86, 95% CI 1.99–178.80, *P* = 0.01). Therefore, referral of these patients to a secondary or tertiary care hospital can be avoided.

A recent study by The GUT and Obesity Asia Workgroup has suggested the utility of 1.45 instead of 1.3 as a low cut‐off value for FIB‐4.^12^ We then re‐evaluated the analysis adopting the 1.45 cut‐off (*n* = 26, 17 advanced fibrosis and 9 without advanced fibrosis) and were able to replicate the findings, which found that a further four patients within the indeterminate group are unlikely to have advanced fibrosis. Serum GGT level above ULN was associated with a 9‐fold (OR 9.38, 95% CI 1.30–67.65, *P* = 0.026) increase in risk, while the presence of diabetes mellitus was associated with a 15‐fold (OR 14.67, 95% CI 1.46–146.96, *P* = 0.022) risk of advanced fibrosis. Further stratification of the patients within the indeterminate group by serum GGT level above ULN and presence of diabetes mellitus could further reduce the number of patients who are unlikely to have advanced fibrosis by 58% (*n* = 15/26) and 42% (n = 11/26), respectively. These findings could enable the decentralized management of patients without advanced fibrosis at the primary care centers.

## Discussion

The major finding of this study was that FIB‐4 score outperforms all other scoring systems for the detection of advanced fibrosis in the Malaysian cohort of NAFLD patients. FIB‐4 score yielded the best AUROC (0.86), specificity (84.7%), and NPV (94%). It also allowed 72% of the patients to avoid further testing, such as transient elastography or liver biopsy, with only 6% patients being misclassified, and a low number of patients fell in the indeterminate group. We also showed, for the first time, that using a low cut‐off value of 1.3 for FIB‐4, further stratification of the patients within the indeterminate group by serum GGT level above ULN and presence of diabetes mellitus could help 52% (*n* = 16/31) and 35% (*n* = 11/31), respectively, of the patients from being referred to secondary/tertiary care hospital.

The increasing NAFLD burden warrants resource‐adaptive management strategies in a community setting. As primary care providers are often the first point of medical contact for patients with or at risk for NAFLD, a simple management algorithm to assist in differentiated care or triage strategy is crucial to assess the level of care needs and timely specialist referral for those at high risk of advanced liver disease. In the absence of an established treatment, the purpose of ruling out advanced fibrosis in NAFLD patients, particularly in the primary care or community setting, is to avoid referring patients who have a lower risk of advanced liver disease to specialists in a hospital setting for further assessment, such as liver biopsy or transient elastography. The increasing number of NALFD patients being referred to liver clinics calls for the assessment of these noninvasive tests to substantially reduce the number of patients being referred, in accordance with the European Association for the Study of the Liver (EASL) Clinical Practice Guidelines, which endorse the use of simple noninvasive methods as the first‐line triage to stratify the risk of advanced fibrosis or cirrhosis.^20^

In this study, all scoring systems had a high NPV (85–94%), indicating that these scoring systems have the accuracy to be used clinically to exclude advanced fibrosis. In contrast to the NPV, each test's PPV was modest, ranging from 32% to 56%. The AUROC ranged from 0.69 to 0.86. Our study revealed that FIB‐4 score provides the highest diagnostic performance; not only does it have the best AUROC (0.86) and NPV (94%), it also rules out advanced fibrosis in 72% of the patients with the lowest false negative result (6%). We found that NFS is slightly inferior to FIB‐4 score. Our findings were supported by a recent meta‐analysis^21^ and a large cohort study,^22^ which reported the highest diagnostic performance with FIB‐4 score followed by NFS.

**Figure 3:**
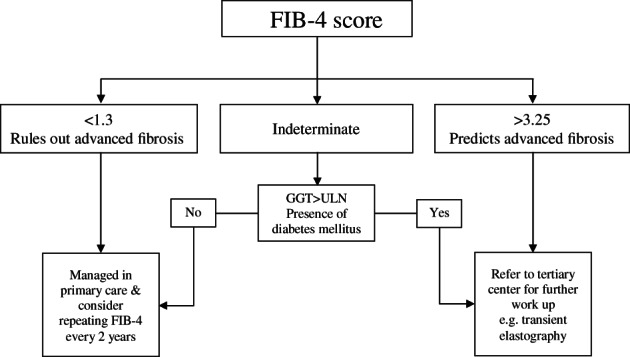
Proposed algorithm for noninvasive assessment.

This study provides a new pragmatic approach to identifying NAFLD patients with a low risk of advanced fibrosis who can be managed in the primary care or community setting using a two‐step approach (Fig. 3). Noninvasive scores such as FIB‐4 can be used as a first step by the primary care providers to assign individuals with NAFLD to one of the three risk categories. Many investigators use the clinical scoring systems to focus mainly on subjects below the lower cut‐off and above the higher cut‐off values, and only few studies evaluate the indeterminate risk group of patients. One study reported the frequency of patients with advanced fibrosis in the indeterminate risk group but did not further stratify the patients.^23^ Several studies, including us, found that this indeterminate or intermediate group of patients represents 15–30% of the total NAFLD patients,^23^, ^24^ and this number could be much higher in the primary care setting involving the general population. EASL Clinical Practice Guidelines on the management of NAFLD recommend further tests such as FibroTest, enhanced liver fibrosis (ELF), or transient elastography to further assess the liver disease status in this group. However, these tests are not available in the primary care setting; hence, clinical parameters such as diabetes status or simple blood test such as GGT can further stratify the “intermediate” risk patients.

We demonstrated that further stratification of the indeterminate patients by other NASH clinical predictors such as serum GGT level and presence of diabetes mellitus can reduce the need for referral of a substantial proportion of patients to tertiary centers.^18^ Our results showed that FIB‐4 score had a quarter of the total number of individuals with NAFLD within the indeterminate range (25%) and with the lowest false negative results (6%). This will translate to a considerable number of individuals with NAFLD in the community setting who need referral to a tertiary center for further workup. Serum GGT level above ULN and presence of diabetes mellitus accounted for 13‐fold and 5‐fold risk of advanced fibrosis, respectively. Chronic hyperglycemia causes a deleterious effect on insulin action and secretion, largely mediated by oxidative stress damaging the pancreatic beta cells, which in turn activates a fibrogenic response.^25^ On the other hand, a study by Lee et al. found that serum GGT is a reliable marker for oxidative stress, especially that of glutathione homeostasis.^26^ In addition, many studies, including from the Gut and Obesity Asia (GO ASIA) Workgroup, showed that diabetes and serum GGT level are independent predictors of advanced fibrosis. Our study suggests that a proportion of patients within the indeterminate group can be further stratified and thus help to reduce the number of some patients from being referred (Fig. 3). Further studies are required to validate the combination of FIB‐4 with clinical predictors of advanced fibrosis to further stratify those with intermediate risk.

One of the limitations of our study is the relatively small sample size. The inclusion of liver biopsy as a criteria for patient recruitment has a limitation in the sample size at one center as liver biopsy is only carried out when there is a definite clinical indication for the biopsy. However, the sample size in our study is similar to several published studies that included biopsy‐proven NAFLD patients.^9^, ^27^, ^28^, ^29^, ^30^, ^31^ Further multi‐institutional studies of larger sample size are necessary for the findings in this study. This study was performed in a tertiary care setting with 20% of the patients with advanced fibrosis. A high degree of advanced fibrosis was similarly seen in 759 biopsy‐proven NAFLD patients (24% with advanced fibrosis) from 10 centers in nine countries in Asia.^19^ The prevalence of NAFLD‐related advanced fibrosis is about 5% in general population.^32^ This may not truly reflect the spectrum of NAFLD patients in the community as a higher proportion is expected to have milder liver disease.

In conclusion, the scoring systems validated in this study were able to noninvasively risk‐stratify patients, thereby identifying those with advance fibrosis or cirrhosis who require specialist referral for additional tests or surveillance and avoiding referral for transient elastrography or liver biopsies in a substantial number of patients without advanced fibrosis. FIB‐4 score outperformed other noninvasive tests in terms of AUROC, NPV, and lower percentage of patients with indeterminate results, in addition to the least number of misclassifications. The FIB‐4 test should be available in the laboratory, and reflex testing for FIB‐4 should be performed for all patients diagnosed with NAFLD and automatically interpreted. Further stratification of patients within the indeterminate range is recommended to avoid a substantial number of patients from being referred, and this can be achieved by assessing the serum GGT level and presence of diabetes. A simplified strategy for a public health approach is needed to decentralize the NAFLD management at the primary care level.
